# Theoretical View: Thermodynamics of the Saturation Dissolution of a Molecular (Solid) Dispersion of a Hydrophobic Molecule and Polymeric Surfactant in an Aqueous Solution

**DOI:** 10.3390/ijms262311756

**Published:** 2025-12-04

**Authors:** Mihalj Poša

**Affiliations:** Department of Pharmacy, Faculty of Medicine, University of Novi Sad, Hajduk Veljka 3, 21000 Novi Sad, Serbia; mihaljp@uns.ac.rs

**Keywords:** hot-melt extrusion, surfactant, micelles, solubilization, Gibbs free energy

## Abstract

Hot-melt extrusion produces a solid dispersion (SD) containing a poorly water-soluble drug (***k***) and matrix polymer surfactant (PS), thereby enhancing ***k***’s solubility. When dissolving the SD, the PS is first dissolved, forming micelles. The amorphous form of the solid phase ***k*** remains and is further dissolved by micellar solubilization. The goal here is to rigorously derive, on the basis of thermodynamics, a new expression for the change in the standard Gibbs free energy (${∆G}_{\Sigma \;}^{0}$). This change serves as a measure for increasing the degree of spontaneity in the dissolution of amorphous ***k*** from an SD with a polymeric surfactant relative to the dissolution of the crystalline-form ***k*** in an aqueous solution without surfactants (reference state). In the micelle-pseudophase model, it was found that ${∆G}_{\Sigma \;}^{0}$ depends on the natural logarithm of the ratio of mole fraction ***k*** in the aqueous phase to mole fraction ***k*** in the micellar pseudophase. In a simpler model, ${∆G}_{\Sigma \;}^{0}$ can be expressed as ln of the solubility ratio of the crystalline and SD forms, assuming that the activity coefficient depends on the process of incorporating ***k*** into the micellar particles and that the total amount of surfactants is many times smaller than the water amount, which is acceptable for polymeric surfactants with low values of the critical micellar concentration.

## 1. Introduction

A rational approach to drug design (pharmacologically active components) usually assumes that the drug molecule has the best possible affinity for the binding site of a biological macromolecule (e.g., a receptor or enzyme). This results in a large number of hydrophobic drug molecules with high affinity but poor solubility in aqueous (physiological) solutions, significantly reducing their bioavailability [1,2]. In formulations used for oral administration, the absorption of drug molecules through the gastrointestinal tract is key, so the pharmacologically active component must be in a soluble form [3]. The solubility of poorly water-soluble drugs can be increased by adding surfactants to their formulations [3,4,5]. Surfactants are amphiphilic molecules which contain separate hydrophobic and hydrophilic molecular regions in their structure [6]. In an aqueous environment at a particular total concentration (a characteristic value for each surfactant)—the critical micellar concentration—surfactants begin to form micellar aggregates whose interior is hydrophobic and exterior is hydrophilic [7,8,9,10,11]. A drug molecule with a hydrophobic segment is incorporated into the hydrophobic core of the micelle. In contrast, the drug’s polar groups are located within the micelle’s hydrophilic shell. Since the micellar aggregate is soluble in water, the incorporated drug is also dissolved, a process known as micellar solubilization [12,13,14]. Formulations of poorly soluble drugs with surfactants can be in either liquid (micellar aqueous solutions) or solid form as a physical mixture of drug and surfactant, whereby the surfactant dissolves in the gastrointestinal tract with the formation of micelles and micellar solubilization of the drug [3]. In addition to surfactants, macrocyclic compounds with a hydrophilic surface and a low-polarity interior can host hydrophobic molecules of low solubility within their cavities, thereby increasing their overall solubility in aqueous media. The most famous are cyclodextrins, whose hydrophobic cavities accommodate the drug’s hydrophobic portions [15,16,17].

To increase the solubility of solid formulations with and without surfactants, hot-melt extrusion (HME) is applied, an advanced pharmaceutical manufacturing process that uses controlled heat and pressure to mix active pharmaceutical ingredients (drugs) with matrix polymers at the molecular level. The HME process results in the formation of amorphous solid dispersions (SDs) that significantly enhance the solubility and bioavailability of drugs, particularly those that are poorly soluble in water. HME operates continuously and is environmentally friendly as it does not use solvents, which can present risks and complications during manufacturing [1,2,3,18,19]. The HME process involves the continuous feeding of a premixed formulation of a drug and matrix polymer (a physical mixture, PM) into an extruder, where the mixture is melted and thoroughly mixed. As the mixture is extruded, it is rapidly cooled to form solid dispersions—molecular-level mixtures. This adaptable method can produce a range of dosage forms, including tablets, films, granules, and miniature pellets, catering to diverse patient needs [18,19,20].

In particular, solid dispersions produced through hot-melt extrusion (HME) technology can be distinguished by the matrix polymer being a polymeric surfactant [3]. In this case, in addition to the amorphous state of the hydrophobic drug in the SD, solubility is also increased by micellar solubilization in the aqueous environment. Polyoxyethylene and polyoxypropylene copolymers (Poloxamers) can be used as polymer surfactants. However, they have a low melting point, and thus in some cases cannot serve as matrix polymers [21]. The polymer surfactant Soluplus^®^ is a graft copolymer composed of polyethylene glycol, polyvinylcaprolactam, and polyvinylacetate, with a significantly higher melting point than Poloxamers, that can be used effectively as both a matrix polymer and surfactant [21,22,23,24]. It is possible to obtain ternary SDs with, in addition to the amorphous drug, a matrix substance (most often a polymer) and a classic non-ionic surfactant or cyclodextrin [20].

The literature typically describes changes in the dissolution rate of SDs relative to the crystalline state of a poorly soluble drug (molecule), i.e., kinetic effects in the solution are studied [1,2,3]. Therefore, the goal here is to use thermodynamic equations to describe the saturation solubility of the amorphous solid phase of a hydrophobic molecule (drug) from a solid solution, when the matrix substance is a polymeric surfactant that forms micelles in aqueous solution. The goal is to obtain the standard change in the Gibbs free energy, which is a measure of the increase in spontaneity in the dissolution of an amorphous drug from SDs with surfactants relative to the process of dissolving a crystalline hydrophobic molecule in an aqueous solution without surfactants and the dissolution of a physical mixture of a hydrophobic molecule and polymeric surfactant (the same amount of surfactant in the SD and physical mixture and the same volume of aqueous solution).

## 2. Results and Discussion

**Figure 1:**
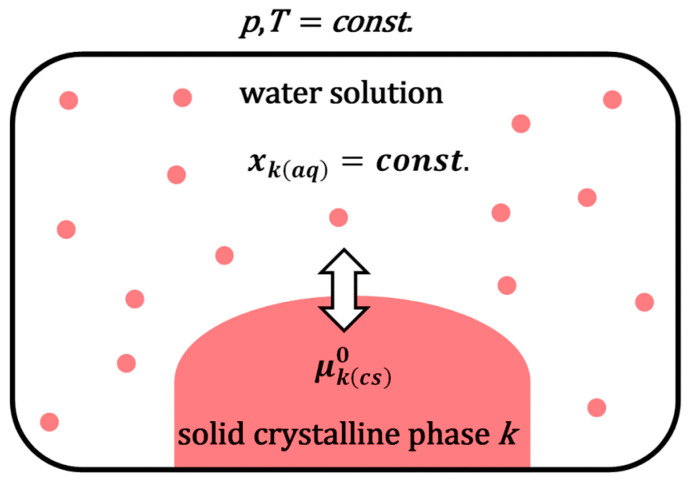
In the presence of a solid phase, equilibrium is established during dissolution; if the solid phase remains present at equilibrium, the process is called saturation solubilization.

Consider a poorly soluble (hydrophobic) molecule ***k*** (drug) in water. Suppose the solid crystalline phase of the hydrophobic molecule ***k*** is in equilibrium with an aqueous solution of the same molecule. In that case, the system simultaneously contains a solid (undissolved) phase of the molecule ***k*** and its infinitely diluted aqueous solution (Figure 1). The chemical potentials of the hydrophobic molecule ***k*** in the undissolved solid crystalline phase and aqueous solution are mutually equal:

(1) $${\mu_{k\left(cs\right)}^{0}=\mu }_{k\left(aq\right)}^{0}+RT\ln x_{k\left(aq\right)}$$

where $\mu_{k\left(cs\right)}^{0}$ is the chemical potential of the molecule ***k*** in the solid crystalline phase (i.e., state, *cs*), and $\mu_{k\left(aq\right)}^{0}$ is the standard (reference) chemical potential of the solubilizate ***k*** in a water solution (the reference state is chosen to be the hypothetical state of the unit mole fraction extrapolated along the Henry’s law line, i.e., from the infinitely diluted aqueous solution of the solubilizate)$.\;x_{k\left(aq\right)}$ represents the solubility (i.e., molar fractions) of ***k*** in a water phase. At constant temperature (*T*) and pressure (*p*), it can be defined as follows [25,26]:

(2) $$x_{k\left(aq\right)}=const.\;if\;p,T=const.$$

According to Equation (1), the standard molar Gibbs free energy (${∆G}_{S(k)}^{0}$) of solubilization ***k*** from the crystalline state is

(3) $${k_{\left(cs\right)}\to k_{\left(aq\right)}:\;∆G}_{S(k)}^{0}=\mu_{k\left(aq\right)}^{0}-\mu_{k\left(cs\right)}^{0}=-RT\ln x_{k\left(aq\right)}$$

Since the solubility of the hydrophobic drug molecule in aqueous solution is low, the activity coefficient in Equation (3) has a value of one in the infinitely (ideally) diluted state of the solution [25,26].

The standard molar Gibbs free energy of the solubilization of a crystalline, poorly water-soluble molecule, ***k*** (${∆G}_{S(k)}^{0}$), is the fundamental reference value for evaluating the efficiency of HME technology in increasing solubility [3,27,28].

**Figure 2:**
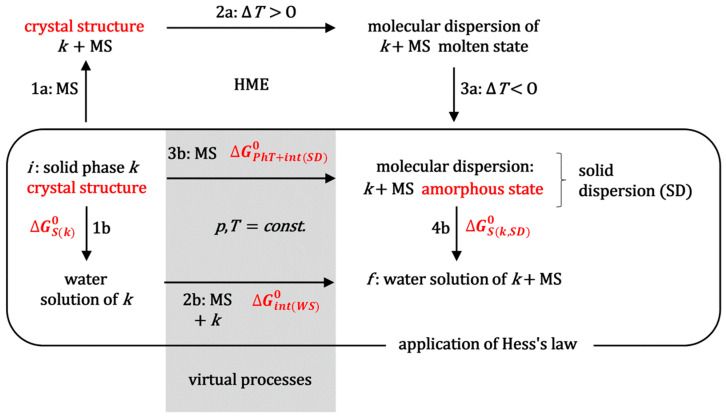
Scheme of the thermodynamic process during the solubilization of the crystalline hydrophobic substance ***k*** and the solubilization of the same substance from the amorphous state of the solid dispersion; processes of obtaining a solid dispersion during HME are also presented (MS = matrix substance, for example, polymeric surfactants, SD = solid dispersion, *i* = initial state, *f* = final state, HME = hot-melt extrusion).

The standard molar Gibbs energy, which indicates the efficiency of hot-melt extrusion technology in enhancing the solubilization of the hydrophobic molecule ***k*** during the saturation experiments, can be determined by applying Hess’s law [29] to the thermodynamic process outlined in Figure 2. This figure illustrates the simultaneous non-spontaneous thermodynamic process involved in HME, specifically the transformation of a crystalline substance (***k***) into the amorphous state within a solid dispersion [1,2]. Process 1a (Figure 2) represents obtaining a physical mixture between the powder of crystalline substance ***k*** and the matrix substance (MS). Let the MS be a block copolymer surfactant, MS = PS, e.g., a polymer from the Poloxamer group, i.e., a polymer that forms micellar aggregates in aqueous solution.

**Figure 3:**
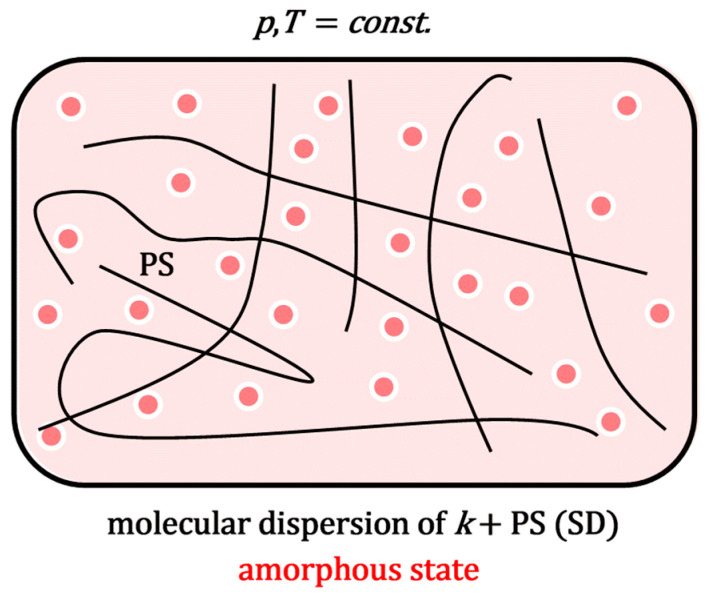
An amorphous solid solution of a polymeric surfactant and a poorly soluble molecule ***k***: mixing is on a molecular scale.

During process 2a (Figure 2), the system (extruder) is heated to above the melting point of the polymeric surfactant, while, with mixing, a molecular dispersion is formed in the molten state. In process 3a, the melt forms an amorphous solid solution upon cooling (SD, Figure 3).

**Figure 4:**
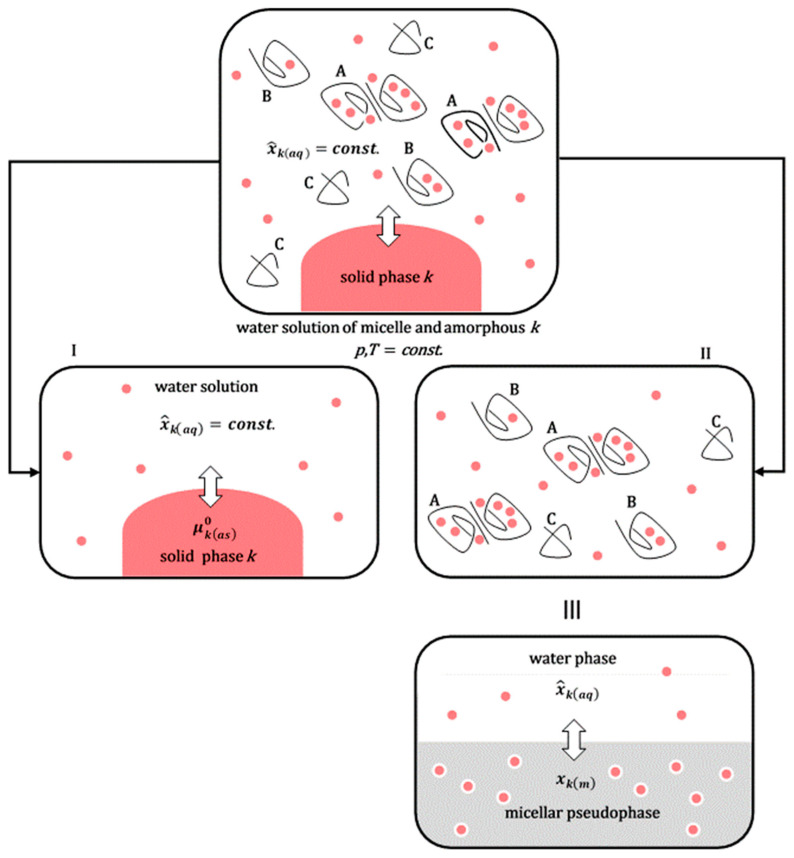
The solubilization of the amorphous solid phase of the hydrophobic molecule ***k*** in the micellar aqueous solution of the polymeric surfactant can be viewed as the sum of the process of solubilization of the amorphous solid phase in the aqueous solution without surfactant (**I**) and the distribution of hydrophobic solute ***k*** between the aqueous solution and micellar aggregates (**II**): micelles with solubilizates ***k*** (A), polymeric surfactants in an unassociated state to whose hydrophobic regions solubilizate ***k*** can also bind (B). There are also shorter polymer chains (C) without solubilizates (polymeric surfactants exhibit polydispersity in polymer chain length [33]; (**III**) two graphical representations of an identical physical state).

When comparing the saturation solubilization of the solid crystalline substance ***k*** (process 1b, Figure 2) with the saturation solubilization of an amorphous solid dispersion (SD) of ***k*** (process 4b, Figure 2), the temperature (*T*), pressure (*p*), and characteristics of the aqueous solution (pH and ionic strength) are the same for both processes. The change in the standard molar Gibbs free energy for process 1b (${∆G}_{S(k)}^{0}$) is described by Equation (3). In process 4b, during the dissolution of the amorphous SD, polymeric surfactants first dissolve in the aqueous solution. Upon reaching a critical micellar concentration, these surfactants form micellar aggregates. These aggregates then incorporate hydrophobic molecules ***k*** into their hydrophobic interiors (Figure 4) [21]. However, process 4b can be represented as the sum of the dissolution process of the amorphous (solid) phase ***k*** (without polymer surfactants) in the aqueous solution and the distribution process of hydrophobic molecules ***k*** between the aqueous phase and micellar aggregates; the last process in the thermodynamic model is viewed as the distribution of ***k*** between the aqueous phase and micellar pseudophase [14].

The change in the standard molar Gibbs free energy (${∆\hat{G}}_{S(k)}^{0}$) for the dissolution of the amorphous solid phase of molecules ***k*** in an aqueous solution is obtained in an analogous way to Equation (3) (Appendix A):

(4) $${k_{\left(as\right)}\to k_{\left(aq\right)}:\;∆\hat{G}}_{S(k)}^{0}=\mu_{k\left(aq\right)}^{0}-\mu_{k\left(as\right)}^{0}=-RT\ln{\hat{x}}_{k\left(aq\right)}$$

where ${\hat{x}}_{k\left(aq\right)}$ represents the mole fraction (solubility) of solute (i.e., solubilizate) in aqueous solution when dissolving the amorphous solid phase of ***k***. However, the chemical potentials of ***k*** molecules in the crystalline (*cs*) and amorphous state (*as*) differ from each other:

(5) $$\mu_{k\left(as\right)}^{0}\neq \mu_{k\left(cs\right)}^{0}$$

Therefore, it follows that the changes in the standard molar Gibbs free energies of dissolution, as well as the solubility values, also differ:

(6) $${∆\hat{G}}_{S(k)}^{0}\neq {∆G}_{S(k)}^{0}\mathrm{and}\;{\hat{x}}_{k\left(aq\right)}\neq x_{k\left(aq\right)}$$

In an aqueous solution of polymer micelles and hydrophobic molecules ***k*** during the distribution of ***k*** between micellar particles and water, equilibrium is established, whereby the set of micellar particles is viewed as a separate phase (micellar pseudophase [30]) where hydrophobic molecules ***k*** have $\mu_{k\left(m\right)}$ chemical potential, i.e., $\mu_{k\left(m\right)}^{0}$ standard chemical potential. Simultaneously, $x_{k\left(m\right)}$ is the mole fraction of solubilizates ***k*** in the micellar pseudophase (Figure 4).

(7) $$\mu_{k\left(aq\right)}=\mu_{k\left(m\right)}\equiv \mu_{k\left(aq\right)}^{0}+RT\ln{\hat{x}}_{k\left(aq\right)}=\mu_{i\left(m\right)}^{0}+RT\ln x_{k\left(m\right)}$$

(8) $${∆G}_{P(k)}^{0}=\mu_{k\left(m\right)}^{0}-\mu_{k\left(aq\right)}^{0}=-RT\ln\left(x_{k\left(m\right)}/{\hat{x}}_{k\left(aq\right)}\right)$$

Equation (8) represents the change in the standard molar Gibbs free energy that follows the distribution (partition) of one mole of hydrophobic particles ***k*** between the micellar and aqueous phases. As ${∆G}_{P(k)}^{0}=const.$ at constant temperature and pressure, for the mole fraction ***k*** from the aqueous phase (generally from any of the two), the value of ${\hat{x}}_{k\left(aq\right)}$ defined by Equation (4) can be arbitrarily taken, whereby the value of $x_{k\left(m\right)}$ is then adjusted to the value of ${\hat{x}}_{k\left(aq\right)}$ to obtain a constant value for ${∆G}_{P(k)}^{0}$. According to the detailed balance theory [31,32], the change in the standard molar Gibbs free energy ${∆G}_{S(k,SD)}^{0}$ (process 4b, Figure 2) can be represented as the sum of the Gibbs free energies of the elementary processes that make up process 4b (solubilization of the amorphous solid ***k*** and the distribution of particles ***k*** between the micellar pseudophase and aqueous phase [33], Figure 4):

(9) $${∆G}_{S(k,SD)}^{0}={∆\hat{G}}_{S(k)}^{0}+{∆G}_{P(k)}^{0}$$

By introducing Equations (4) and (8) into (9), we obtain

(10) $${∆G}_{S(k,SD)}^{0}=-RT\ln{\hat{x}}_{k\left(aq\right)}-RT\ln\left(x_{k\left(m\right)}/{\hat{x}}_{k\left(aq\right)}\right)=-RT\ln x_{k\left(m\right)}$$

In order to apply Hess’s law (Figure 2) between the final (*f*) and initial state (*i*), in addition to the real solubilization processes 1b and 4b, it is necessary to introduce the imaginary processes 2b and 3b (Figure 2). In the imaginary process 2b, a polymeric surfactant and solubilizate ***k*** are introduced into a saturated aqueous solution of hydrophobic solubilizate ***k*** in order to obtain the final state, which is obtained during the solubilization of a solid dispersion (SD) of hydrophobic molecule ***k*** (in an amorphous state) and polymeric surfactant (***k*** is introduced from the aqueous solution where it is in equilibrium with the imaginary amorphous form whose solubility is many times higher than the real amorphous state from the SD, so that there is an imaginary state where the concentration of solubilizate ***k*** is many times higher than in the final state; this way hydrated particles ***k*** are provided). The change in the standard molar Gibbs energy (${∆G}_{int(WS)}^{0}$) in the imaginary process 2b represents the change in the Gibbs free energy due to intermolecular interactions between hydrophobic particles ***k*** and polymer surfactants (mostly) in the micellar state (if in the final state, the concentration of the polymer surfactant is higher than the critical micellar concentration). Since the incorporation of hydrophobic solute into the hydrophobic core of the micelle is an (irreversible) spontaneous process with an entropic driving force around a temperature of 298.15 K (dehydration of the hydrophobic molecular surfaces of the solute ***k*** and the departure of water molecules from the hydration shell above the hydrophobic surface into the interior of the aqueous solution—the molar entropy of the water molecules inside the solution is greater than the molar entropy of the water in the hydration layer above the hydrophobic surface) [34], it can be defined as

(11) $${∆G}_{int(WS)}^{0}<0$$

In the imaginary process 3b (Figure 2), at the same temperature as the initial state, the incorporation of the polymer surfactant destroys the crystal structure. It forms an amorphous state of molecular dispersion. Since during a relatively long time interval, the amorphous SD phase decomposes into the crystalline phase of the hydrophobic molecule ***k*** (initial state) and the solid phase of the polymeric surfactant [3], it follows that in the amorphous SD state the hydrophobic molecule ***k*** is in a thermodynamically less stable state than in the initial crystalline state, despite the formation of molecular dispersion (the Gibbs free energy of the formation of an ideal dispersion is always less than zero [29,31]) and possible intermolecular interactions between the polymeric surfactant and particles ***k***. It further follows that the change in the standard molar Gibbs free energy for the imaginary process 3b (phase transformation: crystalline state → amorphous state (*PhT*) and intermolecular interactions in SD with polymeric surfactants or with the matrix in general (*int*(*SD*))

(12) $${∆G}_{PhT+int(SD)}^{0}>0$$

is valid, i.e., ${∆G}_{PhT+int(SD)}^{0}$ represents the thermodynamic destabilization of the hydrophobic molecule ***k*** in the amorphous SD state in relation to the initial crystalline state.

According to Hess’s law, the change in Gibbs free energy along the path that includes processes 1b and 2b is equal to the change in Gibbs energy along the path that includes processes 3b and 4b if both paths connect the same initial and final state (Figure 2):

(13) $$\underset{processes\;1b+2b}{\underbrace{{∆G}_{S(k)}^{0}+{∆G}_{int(WS)}^{0}}}=\underset{processes\;3b+4b}{\underbrace{{∆G}_{PhT+int(SD)}^{0}+{∆G}_{S(k,SD)}^{0}}}$$

(14) $${∆G}_{\Sigma \;}^{0}={∆G}_{int(WS)}^{0}-{∆G}_{PhT+int(SD)}^{0}={∆G}_{S\left(k,SD\right)}^{0}-{∆G}_{S(k)}^{0}$$

In Equation (14), ${∆G}_{\Sigma \;}^{0}$ represents the standard molar Gibbs free energy as a measure for increasing the degree of spontaneity in the solubilization of hydrophobic molecules ***k*** from the amorphous SD relative to the solubilization of crystalline ***k*** (referent state). By introducing Equations (3) and (10) into (4), we obtain

(15) $${∆G}_{\Sigma \;}^{0}={∆G}_{int(WS)}^{0}-{∆G}_{PhT+int(SD)}^{0}=-RT\ln x_{k\left(m\right)}+RT\ln x_{k\left(aq\right)}=RT\ln\left(x_{k\left(aq\right)}/x_{k\left(m\right)}\right)$$

If after HME it can be proven by structural instrumental methods that polymeric surfactants (PSs) from SDs do not change their aggregation abilities (i.e., no conformational changes occurred in the PS during HME) and if the total amount of PS from the SD dissolves in an aqueous solution, then knowing the values of the critical micellar concentration and the total concentration of the surfactant in the aqueous solution (i.e., how many times the total concentration is greater than the critical micellar concentration), $x_{k\left(m\right)}$ from Equation (15) can be expressed with the molar solubilization ratio (MSR, Appendix B) [26]. Simultaneously, $x_{k\left(aq\right)}$, from Equation (15), can be expressed in terms of the solubility value ($S_{k}$, in molar concentration) of the solubilizate ***k*** in aqueous solution (without polymeric surfactants, Appendix B). Accordingly, Equation (16) is

(16) $${∆G}_{\Sigma \;}^{0}=RT\ln\left(x_{k\left(aq\right)}/x_{k\left(m\right)}\right)=RT\ln\left(\left(\left(MSR+1\right)S_{k}\right)/55.55MSR\right)$$

**Figure 5:**
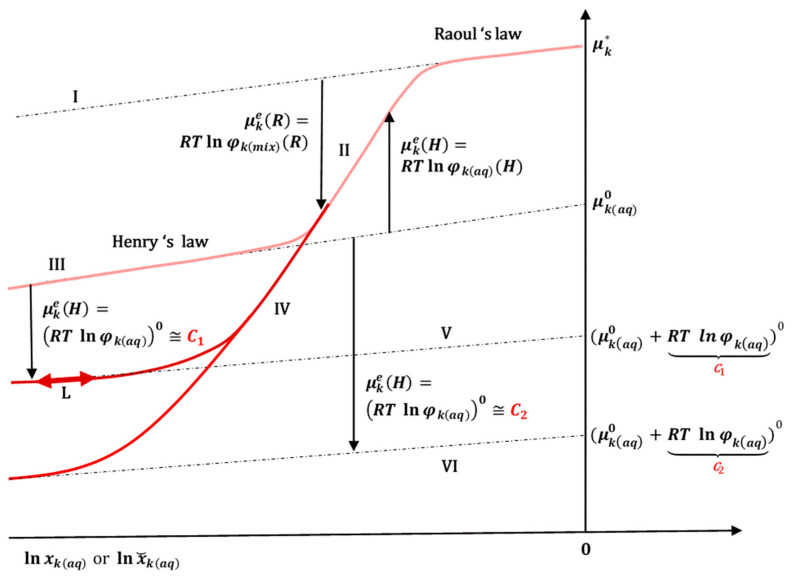
The chemical potential (**I**) that obeys Raoul’s law (the ideal chemical potential that in mixtures linearly depends on lnx) for the reference state has the chemical potential in the pure state of the molecule ***k*** ($\mu_{k}^{*}$); chemical potential (**II**) that deviates from Raoul’s law, i.e., from the linear dependence of the chemical potential on ln*x*, whereby the deviation is described by the excess chemical potential $\mu_{k}^{e}(R)$; in the infinitely (ideally) diluted region, the chemical potential of ln*x* again becomes linear (**III**–**VI**) with the imaginary reference states ($\mu_{k\left(aq\right)}^{0}$ and the new reference states: ${\left(\mu_{k\left(aq\right)}^{0}+RT\ln\varphi_{k\left(aq\right)}\right)}^{0}$. The deviation from the linear Henry’s regime describes the excess chemical potential $\mu_{k}^{e}(H)$—therefore, the same state of the real chemical potential (**II**) can be presented in relation to the reference state $\mu_{k}^{*}$ as well as in relation to the reference state $\mu_{k\left(aq\right)}^{0}$ by applying the appropriate activity coefficients ($\varphi_{k\left(mix\right)}(R)$ or $\varphi_{k\left(aq\right)}(H)$).

**Figure 6:**
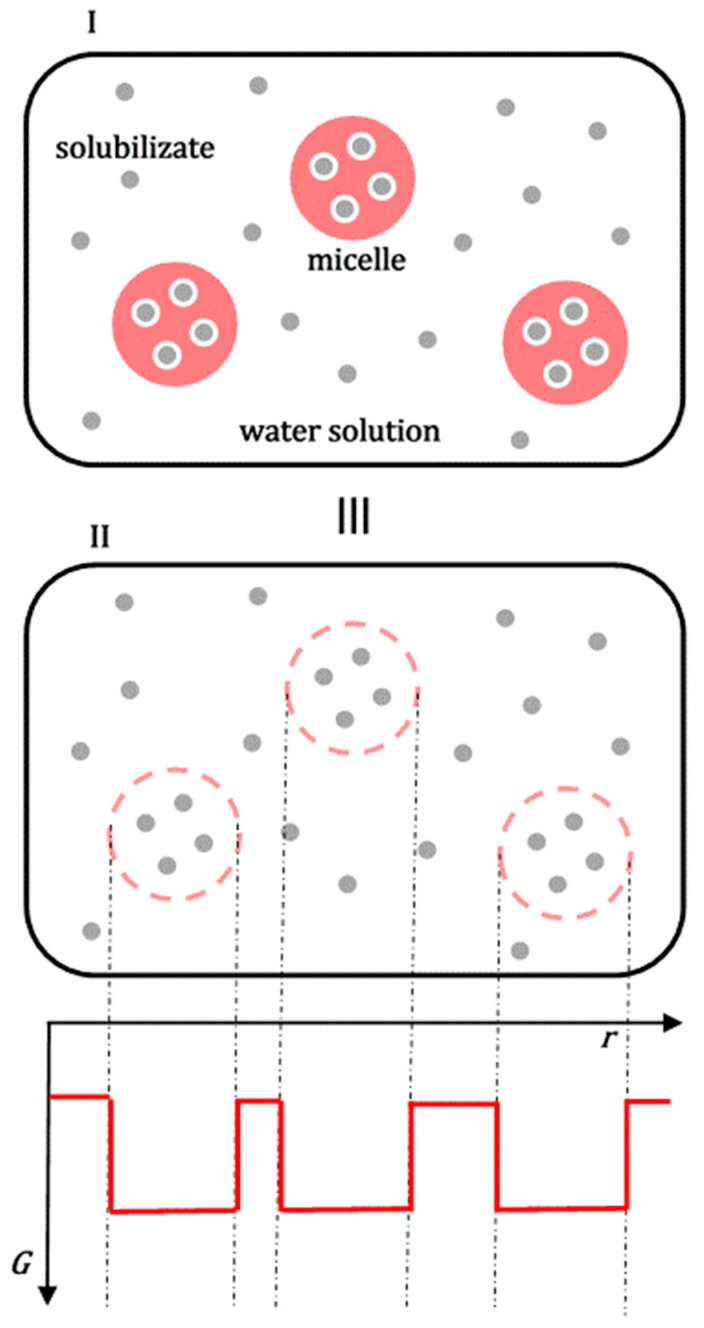
In an aqueous micellar solution of polymeric surfactants, solubilizates are incorporated into micellar aggregates (I), which results in a decrease in Gibbs free energy. Let us look at an imaginary aqueous solution of solubilizate (II), where instead of real micelles, there are spatial locations where solubilizates are grouped (analogously as in micelles). In those spatial environments, they have a lower Gibbs free energy than the environment’s Gibbs free energy; (III) two graphical representations of an identical physical state.

If the critical micellar concentration is not known for the PS or during HME, the aggregation behavior of the polymeric surfactant changes, so the MSR values cannot be determined, and it is necessary to determine $x_{k\left(m\right)}$ experimentally. However, there is a difficulty in determining the mole fraction of the hydrophobic solubilizate ***k*** in the micellar pseudophase experimentally. Namely, during the determination of the solubilizate in micellar solubilization, the micelles are destroyed (by dilution). The total amount of solute ***k*** is determined together with the amount that was already present in the water, in addition to the micellar solubilized amount (the micellar pseudophase, i.e., the set of all micelles could be determined (separated from the aqueous solution) by gel filtration or ultracentrifuge if the micelles are large enough). Therefore, for practical reasons, it is more convenient to avoid the micellar pseudophase and to observe the equilibrium between the hydrophobic molecule ***k*** in the amorphous state (*as*) and the hydrophobic molecule ***k*** from the aqueous micellar solution with mole fraction ${\overset{˘}{x}}_{k\left(aq\right)}$ (it refers to the micellar aqueous solution of the solubilizate and not only the micellar pseudophase and is calculated as the ratio of the number of moles of solubilizate ***k*** ($n_{k}$) to the number of water moles ($n_{w}$) from the aqueous micellar phase ($n_{w}\gg n_{k}$), while the amount of polymeric surfactants is not taken into account). The activity coefficient ($\varphi_{k\left(aq\right)}$) describes the interactions between the solubilizate ***k*** and the micellar aggregates or amphiphilic polymers [25,26]; in this imaginary model, micelles represent Gibbs free energy points, i.e., centers of intermolecular and hydrophobic interactions (Figure 5 and Figure 6):


(17) $$\mu_{k\left(as\right)}^{0}=\mu_{k\left(aq\right)}=\underset{\mu_{k(aq)}}{\underbrace{\mu_{k\left(aq\right)}^{0}+RT\ln{\overset{˘}{x}}_{k\left(aq\right)}+\overset{\mu_{k}^{e}(H)}{\overbrace{RT\ln\varphi_{k\left(aq\right)}}}}}$$

If we take the approximation that the excess chemical potential $\mu_{k}^{e}=\mu_{k}^{e}(H)$ is constant in a diluted micellar solution of solubilizate ***k*** (Figure 5) [35]—the total concentration of the polymeric surfactant (PS) is constant (above the critical micellar concentration)—then for every constant total concentration of PS there is a constant $C_{n}$:

(18) $${\left(RT\ln\varphi_{k\left(aq\right)}\right)}^{0}≅C_{n}$$

from which follows a new imaginary reference state in the chemical potential that obeys Henry’s law (17) (Figure 5 and Appendix C):

(19) $${\left(\mu_{k\left(aq\right)}^{0}+\underset{C_{n}}{\underbrace{RT\ln\varphi_{k\left(aq\right)}}}\right)}^{0}$$

In the equilibrium state, ${\overset{˘}{x}}_{k\left(aq\right)}$ can fluctuates according to the linear function: $\mu_{k\left(aq\right)}={\left(\mu_{k\left(aq\right)}^{0}+RT\ln\varphi_{k\left(aq\right)}\right)}^{0}+RT\ln{\overset{˘}{x}}_{k\left(aq\right)}$ (Figure 5, L). Polymeric surfactants from the Pluronic Block Copolymer group usually have critical micellar concentrations in the range of 0.001 mM to 0.1 mM [36], so that they form micelles in the region of the infinite dilute solution and intermolecular interactions between the micelles themselves can be neglected.

Taking into account Equations (17) and (19) for the chemical potential, the change in the standard molar Gibbs free energy upon dissolution of the SD is

(20) $${∆\overset{˘}{G}}_{S(k,SD)}^{0}={\left(\mu_{k\left(aq\right)}^{0}+RT\ln\varphi_{k\left(aq\right)}\right)}^{0}-\mu_{k\left(as\right)}^{0}=-RT\ln{\overset{˘}{x}}_{k\left(aq\right)}$$

Applying Hess’s law (13), the molar Gibbs free energy of the change in the degree of spontaneity in the solubilization of the hydrophobic particle ***k*** from the amorphous (SD) state is

(21) $${∆G}_{\Sigma \;}^{0}={∆G}_{int(WS)}^{0}-{∆G}_{PhT+int\left(SD\right)}^{0}={∆\overset{˘}{G}}_{S(k,SD)}^{0}-{∆G}_{S\left(k\right)}^{0}=-RT\ln{\overset{˘}{x}}_{k\left(aq\right)}+RT\ln x_{k\left(aq\right)}=RT\ln\left(x_{k\left(aq\right)}/{\overset{˘}{x}}_{k\left(aq\right)}\right)$$

**Figure 7:**
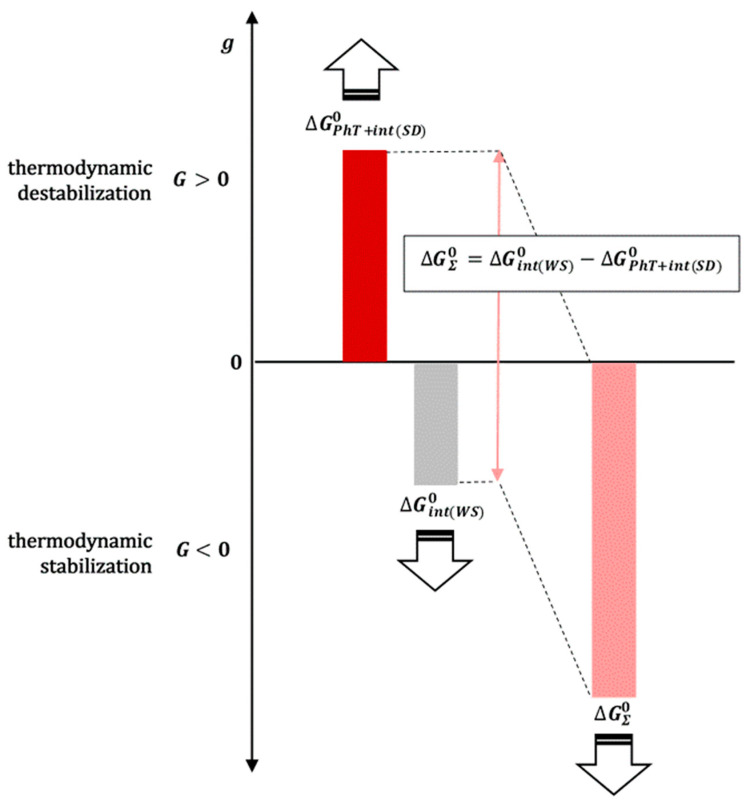
The solubilization of the hydrophobic particle ***k*** from the amorphous state is all the more spontaneous if its thermodynamic destabilization in relation to the crystalline state is greater, and at the same time, if its thermodynamic stabilization in an aqueous solution of additives (micellar solution of polymeric surfactants) is greater than in the state of an aqueous solution without additives.

If the ratio of mole fractions in Equation (21) is

$${\overset{˘}{x}}_{k\left(aq\right)}>x_{k\left(aq\right)}$$

then ${∆G}_{\Sigma \;}^{0}<0$, which means that the degree of spontaneity increases during the solubilization of amorphous ***k*** from the SD in relation to the solubilization of crystalline ***k***. From Equation (21), it can be concluded that ${∆G}_{\Sigma \;}^{0}$ is more negative if ${∆G}_{PhT+int(SD)}^{0}>0$ and has the highest possible value, and also if ${∆G}_{int(WS)}^{0}<0$ and has the highest possible absolute value. Therefore, ${∆G}_{\Sigma \;}^{0}$ is as negative as possible if the hydrophobic molecule ***k*** in the amorphous state has a higher energy content than in the crystalline (reference) state, i.e., is more thermodynamically destabilized, and if there are additional intermolecular interactions (and/or hydrophobic effect) between the solubilizate ***k*** and the amphiphilic polymers or the micellar polymer surfactants in the aqueous solution, the solubilizate ***k*** is in a thermodynamically more stable state than in pure water (Figure 7).

**Figure 8:**
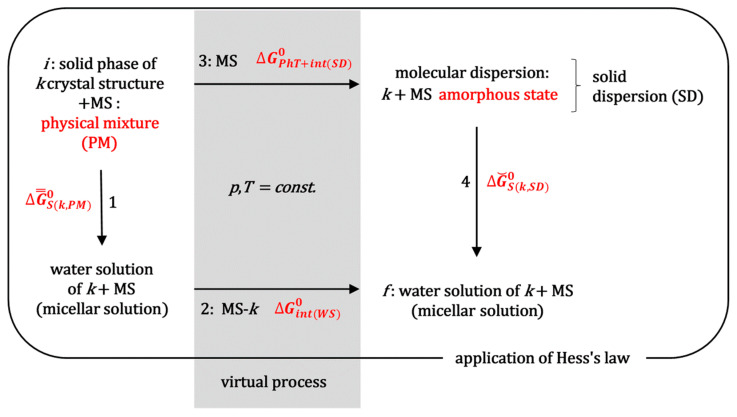
The reference (initial, *i*) state is a physical mixture (PM) of a crystalline hydrophobic substance ***k*** and matrix substance (MS), which, for example, can be a polymeric surfactant MS = PS; PM and SD contain the same amount of matrix substance (i.e., polymeric surfactant), therefore the aqueous solution in both cases (processes 1 and 4) contains the same amount of polymeric surfactant. In the imaginary process 2, the hydrated molecules ***k*** are incorporated into micelles to obtain the final state, so that ${∆G}_{int(WS)}^{0}$ is the change in the molar Gibbs free energy that follows the incorporation of ***k*** into polymeric micelles (*f* = final state, SD = solid dispersion obtained by the HME process).

However, the question can be raised as to whether the thermodynamic destabilization of the molecule ***k*** in the amorphous state compared to the (reference) crystalline state is more important to the values of ${∆G}_{\Sigma \;}^{0}$ than the thermodynamic stabilization of the hydrophobic solubilizate ***k*** in the aqueous solution of micellar polymeric surfactants (or amphiphilic polymers) [3]. Therefore, it is necessary to observe the solubility of crystalline ***k*** from a physical mixture (PM) with a matrix substance (amphiphilic polymers or polymeric surfactants). As the matrix polymers are soluble in water, they dissolve first. Suppose the polymer surfactants are present and their concentration is above the critical micellar concentration. In that case, they form micelles, so that the crystalline substance ***k*** dissolves in the micellar solution of the polymeric surfactant (process 1, Figure 8). Applying equilibrium conditions, i.e., the equality between the chemical potential of ***k*** in the crystalline solid state and the chemical potential of ***k*** in the micellar aqueous solution (or solution of amphiphilic polymers), leads to a change in the standard molar Gibbs free energy in process 1 (Figure 8), analogous to Equation (20):

(22) $${∆\overset{̿}{G}}_{S(k,PM)}^{0}={\left(\mu_{k\left(aq\right)}^{0}+RT\ln\varphi_{k\left(aq\right)}\right)}^{0}-\mu_{k\left(cs\right)}^{0}=-RT\ln{\overset{̿}{x}}_{k\left(aq\right)}$$

where ${\overset{̿}{x}}_{k\left(aq\right)}$ represents the mole fraction of solubilizate ***k*** in aqueous solution when dissolving the physical mixture (PM). Figure 8 presents a scheme of the thermodynamic processes (including the dissolution of a physical mixture) whose changes in standard molar Gibbs free energies are defined in the thermodynamic scheme from Figure 2. They are defined by the following expressions: process 2 (11), process 3 (12), and process 4 (20). Applying Hess’s law to the thermodynamic scheme in Figure 2, we obtain

(23) $$\underset{processes\;1b+2b}{\underbrace{{∆\overset{̿}{G}}_{S(k,PM)}^{0}+{∆G}_{int(WS)}^{0}}}=\underset{processes\;3b+4b}{\underbrace{{∆G}_{PhT+int(SD)}^{0}+{∆\overset{˘}{G}}_{S(k,SD)}^{0}}}$$

(24) $${∆G}_{\Sigma \;}^{0}={∆G}_{int(WS)}^{0}-{∆G}_{PhT+int(SD)}^{0}={∆\overset{˘}{G}}_{S(k,SD)}^{0}-{∆\overset{̿}{G}}_{S(k,PM)}^{0}$$

By introducing Equations (20) and (22), the following expression is obtained:

(25) $${∆G}_{\Sigma \;}^{0}=-RT\ln{\overset{˘}{x}}_{k\left(aq\right)}+RT\ln{\overset{̿}{x}}_{k\left(aq\right)}=RT\ln\left({\overset{̿}{x}}_{k\left(aq\right)}/{\overset{˘}{x}}_{k\left(aq\right)}\right)$$

If in Equation (25), ${\overset{˘}{x}}_{k\left(aq\right)}>{\overset{̿}{x}}_{k\left(aq\right)}$; then, ${∆G}_{\Sigma \;}^{0}<0$ applies, which means that the dissolution of the amorphous solid phase of molecules ***k*** in a micellar aqueous solution of polymeric surfactants is a more thermodynamically spontaneous process than the dissolution of a crystalline substance ***k*** from a physical mixture in the same micellar solution, i.e., the thermodynamic destabilization of the particle ***k*** in the amorphous state relative to the particle ***k*** from the crystalline structure is the leading phenomenon that increases the solubility of ***k***.

In the literature [27,28], there is an empirical equation for ${∆G}_{\Sigma \;}^{0}$ (applied without deriving and proving the equation):

(26) $${∆G}_{tr\;}^{0}=RT\ln\left(S_{k}/S_{k-SD}\right)$$

in which, instead of the mole fractions of hydrophobic molecules ***k***, the solubilities of substance ***k*** per unit volume of aqueous solution are used ($S_{k}$ = the solubility of the crystalline solid phase of ***k*** in water, and $S_{k-SD}$ = the solubility of amorphous ***k*** from a solid dispersion in water). In Equation (21), which was obtained by applying thermodynamic formalism, the mole fractions can be represented as follows ($n_{k}$ or ${\overset{˘}{n}}_{k}=$ amount of substance ***k***; $n_{w}$ = amount of water):

(27) $$x_{k\left(aq\right)}=n_{k}/\left(n_{k}+n_{w}\right)\mathrm{and}\;{\overset{˘}{x}}_{k\left(aq\right)}={\overset{˘}{n}}_{k}/\left({\overset{˘}{n}}_{k}+n_{w}\right)$$

If the observed system is a dilute solution so that conditions $a:\;n_{w}\gg n_{k}$ and $b:\;n_{w}\gg {\overset{˘}{n}}_{k}$ apply, then the mole fractions can be approximated by

(28) $$x_{k\left(aq\right)}≅n_{k}/n_{w},\;{\overset{˘}{x}}_{k\left(aq\right)}≅{\overset{˘}{n}}_{k}/n_{w}$$

and be represented by equations ($m_{k}$ and ${\overset{˘}{m}}_{k}$ = mass of solubilizate ***k***, $M_{k}$ = molecular weight of solubilizate ***k***, $M_{w}$= molecular weight of water, $\vartheta_{w}$= water density, and $V_{w}$ = the volume of aqueous solution which is identical when dissolving crystalline ***k*** and amorphous ***k*** from the SD):

(29) $$x_{k\left(aq\right)}≅\left(m_{k}M_{w}\right)/\left({M_{k}\vartheta }_{w}V_{w}\right),\;{\overset{˘}{x}}_{k\left(aq\right)}≅\left({\overset{˘}{m}}_{k}M_{w}\right)/\left({M_{k}\vartheta }_{w}V_{w}\right)$$

Therefore, ${∆G}_{\Sigma \;}^{0}$ equates to ($S_{k}=m_{k}/V_{w}$ and $S_{k-SD}={\overset{˘}{m}}_{k}/V_{w})$

(30) $${∆G}_{\Sigma \;}^{0}≅RT\ln\left(x_{k\left(aq\right)}/{\overset{˘}{x}}_{k\left(aq\right)}\right)=\left(\left(m_{k}M_{w}\right)/\left({M_{k}\vartheta }_{w}V_{w}\right)/\left({\overset{˘}{m}}_{k}M_{w}\right)/\left({M_{k}\vartheta }_{w}V_{w}\right)\right)$$

(31) $${∆G}_{\Sigma \;}^{0}≅RT\ln\left(m_{k}/{\overset{˘}{m}}_{k}\right)≅{∆G}_{tr\;}^{0}=RT\ln\left(S_{k}/S_{k-SD}\right)$$

If it is true that the solution is diluted, i.e., approximations (28) are acceptable, then it holds that ${∆G}_{\Sigma \;}^{0}$ is approximately equal to ${∆G}_{tr\;}^{0}$. Similar to Equations (25) and (31), they can be presented identically with the solubilities of the hydrophobic substance ***k*** from the physical mixture (PM) and the solid dispersion (SD):

(32) $${∆G}_{\Sigma \;}^{0}=RT\ln\left({\overset{̿}{x}}_{k\left(aq\right)}/{\overset{˘}{x}}_{k\left(aq\right)}\right)≅RT\ln\left({\overset{̿}{m}}_{k}/{\overset{˘}{m}}_{k}\right)=RT\ln\left(S_{k-PM}/S_{k-SD}\right)$$

If in a solid dispersion obtained by HMT technology, the ratio of a poorly water-soluble drug ***k*** and the polymer matrix remains constant; however, the composition within the polymer matrix is modified by adding a non-polymeric surfactant or another polymer surfactant or a polymer that does not build micelles, then the efficiency of solubilization of ***k*** in a solid dispersion with a modified polymer matrix can be compared with the solubilization of ***k*** from a solid dispersion with the reference polymer matrix. Applying Hess’s law to a thermodynamic scheme that is analogous to the schemes in Figure 1 or Figure 8, and where one of the real processes is the reference process of solubilization of amorphous ***k*** from SD with a reference polymer matrix, while the other real process is the solubilization of ***k*** from SD with a changed composition of the polymer matrix, then for ${∆G}_{\Sigma \;}^{0}$ an Equation is obtained that is related to Equation (32):

(33) $${∆G}_{\Sigma \;}^{0}≅RT\ln\left(S_{k-SD:Ref}/S_{k-SD:Mod}\right)$$

where $\;S_{k-SD:Ref}$ represents the solubilization of ***k*** from SD with the reference polymer matrix, while $S_{k-SD:Mod}$ is the solubilization of the same molecule with a modified polymer matrix. To illustrate the values for ${∆G}_{\Sigma \;}^{0}$, Equation (33), Table 1 presents the solubilization values of the drug lornoxicam in an aqueous solution [27], where the reference polymer matrix is the polymer surfactant Soluplus^®^, while the modified polymer matrices of the mixture are: Soluplus^®^-Lutrol F127, Soluplus^®^-Lutrol F68, and Soluplus^®^-polyethylene Glycol 400.

The value ${∆G}_{\Sigma \;}^{0}>0$ means that the process of solubilization of amorphous solid phase ***k*** with a modified polymer matrix (PM) is a thermodynamically less spontaneous process than the dissolution of amorphous ***k*** with a reference polymer matrix. In contrast, ${∆G}_{\Sigma \;}^{0}<0$ means that the dissolution of amorphous ***k*** with a modified PM is a more spontaneous thermodynamic process than the same process with a reference PM.

The saturation solubility experiment is conducted under constant temperature and pressure conditions, with the thermoreservoir serving as the environment. According to De Donder’s equation ($dS_{T}$ = total differential entropy change in the composite system: system and environment, $TdS_{T}=-dG$ [37]), we can derive the following expression (considering Equations (31) and (32)):

(34) $$∆S_{\Sigma \;}^{0}=-∆G_{\Sigma \;}^{0}/T$$

In this context, $-∆G_{\Sigma \;}^{0}/T$ represents the increase in total entropy (for $∆G_{\Sigma \;}^{0}<0$) in the total entropy change of the composite system in which amorphous *k* from SD is dissolved relative to the total entropy change in the reference composite system (dissolution of crystalline *k* or physical mixture).

### 2.1. Dissolution Process as a Chemical Reaction

The differential change in Gibbs free energy is [38]

(35) $$dG=-SdT+Vdp+\sum \mu_{k}dn_{k}$$

that is, at constant temperature and pressure:

(36) $$dG=\sum \mu_{k}dn_{k}$$

Consider the dissolution process as a chemical reaction, i.e., dissolution (transfer) of one mole of ***k*** from the crystalline form into a saturated aqueous solution:

(37) $$k_{\left(cs\right)}\to k_{\left(aq\right)}$$

so that the degree of progress (ξ) in (37) can be defined [39]. The differential change in the amount of molecules in Equation (36) can be expressed by the degree of progress in the reaction and with the stoichiometric number:

(38) $$dG=\left(\sum \mu_{k}\nu_{k}\right)d\xi$$

(39) $${\left(\partial G/\partial \xi \right)}_{p,T}=∆G_{R}=\sum \mu_{k}\nu_{k}$$

Therefore, $∆G_{R}$ represents the slope of the dependence of the Gibbs free energy on the degree of progress in the reaction. In the state of equilibrium (${\xi =\xi }_{e}$), Equation (39) is

(40) $$0=\sum \mu_{k}\nu_{k}=\sum \mu_{k}^{0}\nu_{k}+RT\ln K$$

(41) $$\sum \mu_{k}^{0}\nu_{k}=∆G_{R}^{0}=-RT\ln K$$

where $∆G_{R}^{0}$ represents the standard change in the Gibbs free energy of the reaction, while *K* is the equilibrium constant. Equation (41) for the dissolution process of crystalline ***k*** is identical to Equation (3). Let $∆G_{R}^{0}$ (*p*, *T* = *const.*), in addition to *ξ*, also depend on the parameter *α* [40], which can be a measure for the presence of surfactants in different amounts (above the critical micellar concentration) and (or) the degree of translation of the crystalline form ***k*** into the amorphous form. The total differential for $∆G_{R}^{0}$ as a function of $∆G_{R}=f(\xi ,\alpha )$ is

(42) $$d{\left(∆G_{R}\right)}_{p,T}={\left(\partial ∆G_{R}/\partial \alpha \right)}_{p,T,\xi }d\alpha +{\left(\partial ∆G_{R}/\partial \xi \right)}_{p,T,\alpha }d\xi$$

In a state of equilibrium, it is

(43) $$0={\left(\partial ∆G_{R}/\partial \alpha \right)}_{p,T,\xi }d\alpha +{\left(\partial ∆G_{R}/\partial \xi \right)}_{p,T,\alpha }d\xi$$

Let expression (43) be divided by dα:

(44) $$0={\left(\partial ∆G_{R}/\partial \alpha \right)}_{p,T,\xi }+{\left(\partial ∆G_{R}/\partial \xi \right)}_{p,T,\alpha }\left\{d\xi /d\alpha \right\}$$

(45) $$d\xi /d\alpha =-{\left(\partial ∆G_{R}/\partial \alpha \right)}_{p,T,\xi }{\left(\partial ∆G_{R}/\partial \xi \right)}_{p,T,\alpha }^{-1}$$

Given the definition of $∆G_{R}$ (39), Equation (45) is

(46) $$d\xi /d\alpha =-{\left(\partial \left(\partial G/\partial \xi \right)/\partial \alpha \right)}_{p,T,\xi }{\left(\partial^{2}G/\partial \xi^{2}\right)}_{p,T,\alpha }^{-1}$$

Taking into account the convexity of the function *G* depending on ξ [38,40,41,42], it follows that

(47) $${\left(\partial^{2}G/\partial \xi^{2}\right)}_{p,T,\alpha }>0$$

Therefore, the sign of dξ/dα depends on the sign of ${\left(\partial \left(\partial G/\partial \xi \right)/\partial \alpha \right)}_{p,T,\xi }={\left(\partial ∆G_{R}/\partial \alpha \right)}_{p,T,\xi }$. The slope ${\left(\partial G/\partial \xi \right)}_{p,T}=∆G_{R}$ (39) can be expressed as the ratio of the reaction quotient (*Q*) [29] and the reaction equilibrium constant:

(48) $${\left(\partial G/\partial \xi \right)}_{p,T}=∆G_{R}=\sum \mu_{k}\nu_{k}=\sum \mu_{k}^{0}\nu_{k}+RT\ln Q=-RT\ln K+RT\ln Q=RT\ln\left(Q/K\right)$$

Moreover, since during the reaction progress, it is Q<K that follows $\ln\left(Q/K\right)<0$, i.e., ${\left(\partial G/\partial \xi \right)}_{p,T}<0$. Inserting Equation (48) into Equation (46) yields

(49) $$d\xi /d\alpha =-{RT\left(\partial \left(\ln\left(Q/K\right)\right)/\partial \alpha \right)}_{p,T,\xi }{\left(\partial^{2}G/\partial \xi^{2}\right)}_{p,T,\alpha }^{-1}$$

**Figure 9:**
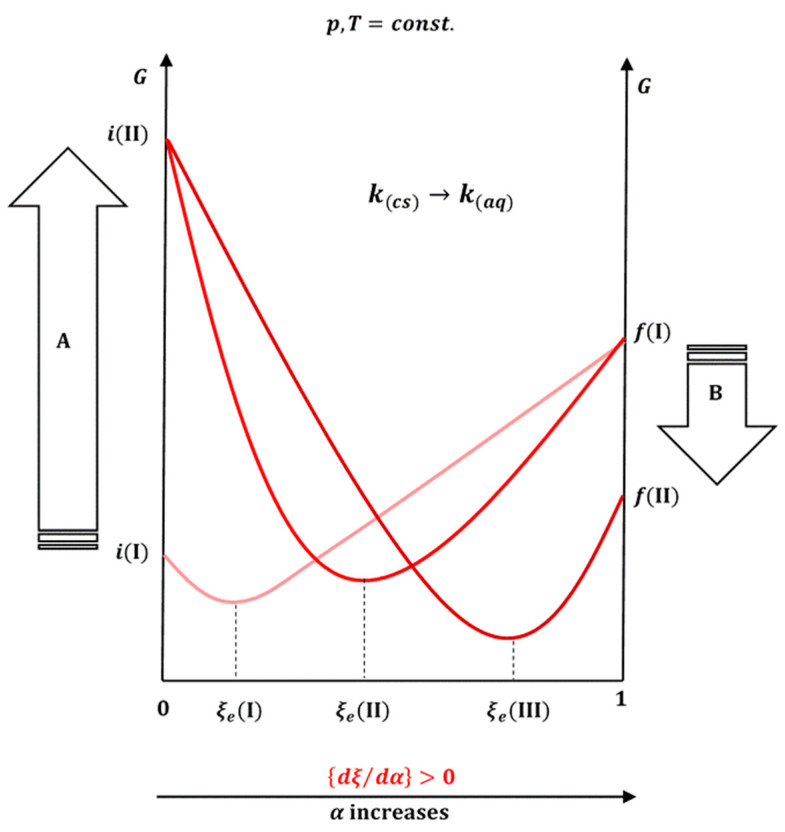
The process of dissolution of the hydrophobic molecule ***k*** from the crystalline (*cs*) and amorphous state if viewed as a chemical reaction. In the initial state, *i*(I), ***k*** is in a crystalline state, and equilibrium is established in the solubilization process at ξ*e*(I). In process A, the crystalline state ***k*** is transformed into the amorphous state *i*(II), which results in a new equilibrium state ξ*e*(II) that is shifted to the right, i.e., increasing solubility. The presence of surfactants in the aqueous solution (B: the process of adding surfactants, in addition to the presence of an amorphous state) changes the final state (*f*), which results in the equilibrium state moving even more to the right, ξ*e*(III).

By converting the crystalline form ***k*** into the amorphous form, the value of *K* increases (the Q/K ratio decreases), which means that with an increase in the proportion of the amorphous form, it is ${\left(\partial \left(\ln\left(Q/K\right)\right)/\partial \alpha \right)}_{p,T,\xi }<0$. Therefore, with an increase in *α*, the degree of progress of the reaction in the new equilibrium state is higher, $\left\{d\xi /d\alpha \right\}>0$, i.e., the solubility of the hydrophobic molecule ***k*** increases. An increase in the total surfactant concentration above the critical micellar concentration has a similar effect (Figure 9).

### 2.2. Future Perspectives

In the dissolution process of the amorphous form of ***k*** from SD with polymeric surfactant (PS), for the purpose of applying thermodynamic models, it is important to determine experimentally whether the dissolution of PS can be considered instantaneous, so that the micellar solubilization of the one-component amorphous solid phase ***k*** is observed. It is important to determine, especially for polymeric surfactants, whether, during the HME technological process (after returning to the initial temperature), there are conformational changes in the polymer chains that would alter the critical micellar concentration. With the applicability of Equations (19) and (20) by molecular dynamic methods, the existence of a linear dependence between the excess chemical potential and ln*x* (infinitely diluted solutions in terms of solubilizates) at different total concentrations of polymeric surfactant (above the critical micellar concentration) could be established. The development of a thermodynamic model includes the activity coefficient which also takes into account intermicellar interactions, especially if the surfactant concentration is several times above the critical micellar concentration.

In HME technology used for obtaining a solid mixture (SD) of a hydrophobic drug and polymer matrix, in addition to polymer surfactants, non-polymeric (classical) surfactants and polymers that do not form micelles on their own could be included. However, suppose surfactants and polymers that form aggregates in aqueous solution are selected; upon dissolving SD, depending on the molar ratio of surfactant to polymer, different aggregates form. In that case, if the polymer chain is already saturated with surfactants, micelles form as well. Therefore, the saturation solubilization of a hydrophobic drug can be determined at different mole ratios of surfactant and polymer [43,44]. Similarly, in HME technology used for obtaining a solid mixture of a hydrophobic drug and polymer matrix, ionic surfactants with a simple counterion and a polyion with a simple counterion could also be included in the polymer matrix. When dissolving a SD, the surfactant ions and polyion (polymer) form a complex salt that can eventually solubilize the amorphous hydrophobic drug [45].

## 3. Materials and Methods

The method of classical thermodynamics applies such that at the equilibrium state of the system, the chemical potentials of component ***k*** are equal in every phase where ***k*** is present [25].

## 4. Conclusions

The increase in spontaneity (${∆G}_{\Sigma \;}^{0}$) of the thermodynamic process of the dissolution of amorphous ***k*** from SDs with polymeric surfactants (PSs) relative to the thermodynamic process of the dissolution of crystalline ***k*** in an aqueous solution (without additives) can be exactly expressed thermodynamically with Equation (15) if the mole fraction of the solubilizate ***k*** in the micellar pseudophase is known, or if the exact concentration and critical micellar concentration of the polymeric surfactant in the aqueous solution are known (application of the MSR parameter). Suppose the above state values of the solubilizate ***k*** are not known. In that case, ${∆G}_{\Sigma \;}^{0}$ can be approximately obtained (Equation (31)) knowing the solubility of amorphous ***k*** from the SD with PS (as well as knowing the solubility of the crystalline form ***k*** in water) by applying the following approximations:

1. There are no interactions between the micellar particles;
2. The intermolecular interactions during the incorporation of the solubilizate ***k*** into the micellar aggregates are contained in the activity coefficient, i.e., in excess chemical potential.

A qualitative description of the effect of the conversion of the crystalline form ***k*** into the amorphous form ***k*** and the presence of micellar particles in the aqueous solution can also be obtained if the thermodynamic process of the dissolution of a poorly soluble molecule is viewed as a chemical reaction.

## Figures and Tables

**Table 1:** Solubility of the drug lornoxicam from a solid dispersion (SD) obtained by HME technology in an aqueous solution in a saturation experiment at a temperature of 37 ± 0.5 °C, and the value of ${∆G}_{\Sigma \;}^{0}$ according to Equation (33).

SD	Lornoxicam-Soluplus^®^ (1:3)	Lornoxicam-Soluplus^®^-Lutrol F127 (1:2.8:0.2)	Lornoxicam-Solup-lus^®^-Lutrol F68 (1:2.8:0.2)	Lornoxicam-Soluplus^®^-Polyethylene Glycol 400 (1:2.8:0.2)
$S_{k-SD:Ref}$ [27]/mg mL^−1^	0.063			
$S_{k-SD:Mod}$ [27] /mg mL^−1^		0.044	0.168	0.105
${∆G}_{\Sigma \;}^{0}$/kJ mol^−1^		0.92	−2.53	−1.32

## Data Availability

The original contributions presented in this study are included in the article. Further inquiries can be directed to the corresponding author.

## Abbreviations

The following abbreviations are used in this manuscript:

HME	hot-melt extrusion
SD	solid dispersions
PM	physical mixture
MS	matrix substances
PS	polymer surfactant
MSR	molar solubilization ratio
CMC	critical micellar concentration
(*cs*)	crystalline solid phase-state
(*as*)	amorphous solid phase-state
(*aq*)	aqueous solution
(*m*)	micellar pseudophase

## Appendix A

Since the amorphous state of the hydrophobic substance ***k*** is generally not an equilibrium state, thermodynamic equilibrium conditions cannot be applied to such a system, i.e., the equality of the chemical potential of ***k*** from the amorphous state and the chemical potential of ***k*** from some other phase. However, due to the relatively long time of transformation of the amorphous state into the crystalline state (actual equilibrium state), according to Calen, the amorphous state is a metastable equilibrium state [38], which means that the transformation is so slow, that during the solubilization process, the amorphous state can be considered constant and apply the conditions of thermodynamic equilibrium (equality of chemical potentials of ***k*** from different phases).

## Appendix B

The ability of a micellar aqueous solution to solubilize poorly soluble particles ***k*** in water is quantified by the molar solubilization ratio (MSR):

(A1) $$MSR=\left(S_{n\times CMC}-S_{CMC}\right)/\left(n\times CMC-CMC\right)\;$$

MSR indicates the number of moles of the solubilizate ***k*** that can be dissolved by one mole of micellar surfactant and *n* represents how many times the total surfactant concentration is greater than the critical micellar concentration. In this context, $S_{CMC}=S_{k}$ represents the solubility of the solubilizate ***k*** at CMC (critical micellar concentration), which is the point at which the surfactant has not yet formed micelles. In contrast, $S_{n\times CMC}$ refers to the solubility of the solubilizate ***k*** when the total surfactant concentration equals the CMC multiplied by *n*, indicating micellar solubilization. If the MSR values are known, then the mole fraction of the solubilized ***k*** in the micellar pseudophase can be calculated. From the definition of MSR, it follows that MSR corresponds to the number of moles of solubilizate ***k*** in the mixture (micellar pseudophase) consisting of 1 mole of surfactant and MSR of moles of solubilizate ***k***, so that the mole fraction of solubilizate ***k*** in the micellar pseudophase is:

(A2) $$x_{k\left(m\right)}=MSR/\left(MSR+1\right)$$

If the solubility $S_{k}$ is expressed in moldm^−3^, then the number of moles of solubilizate ***k*** in an aqueous solution of volume 1 dm^3^ is $S_{k}$. Since the amount of water in the solution is many times greater than the amount of solubilizate ***k***, the approximation is that the total amount of particles equals the amount of water molecules. If the density of water is 1000 gdm^−3^, then 1 dm^3^ of water has a mass of 1000 g, that is, an amount of 55.55 moles. Therefore, the mole fraction of the hydrophobic solubilizate ***k*** in the aqueous phase is:

(A3) $$x_{i\left(aq\right)}=n_{i}/55.55=S_{k}/55.55$$

## Appendix C

In the state of thermodynamic equilibrium during micellar solubilization of amorphous ***k*** (*as*) from SD is (*p*,*T* = *const.*):

(A4) $${\mu_{k\left(as\right)}^{0}=\mu }_{k\left(aq\right)}=\mu_{k\left(m\right)}$$

(A5) $${\mu_{k\left(as\right)}^{0}=\mu_{k\left(aq\right)}=\mu }_{k\left(aq\right)}^{0}+RT\ln x_{k\left(aq\right)}=\mu_{k\left(m\right)}$$

where $\mu_{k\left(as\right)}^{0}$ is the (reference) chemical potentials of ***k*** molecules in the amorphous state (*as*). If, at the equilibrium state (A5), the chemical potential for the solubilizate ***k*** from the aqueous solution is expressed with Equation (16) with the application of definition (18), we get:

(A6) $$\mu_{k\left(as\right)}^{0}=\mu_{k\left(aq\right)}={\left(\mu_{k\left(aq\right)}^{0}+RT\ln\varphi_{k\left(aq\right)}\right)}^{0}+RT\ln{\overset{˘}{x}}_{k\left(aq\right)}$$

However, as equality (A5) holds, expressions (A5) and (A6) can be mutually equalized:

(A7) $$\mu_{k\left(aq\right)}^{0}+RT\ln x_{k\left(aq\right)}={\left(\mu_{k\left(aq\right)}^{0}+RT\ln\varphi_{k\left(aq\right)}\right)}^{0}+RT\ln{\overset{˘}{x}}_{k\left(aq\right)}$$

(A8) $$RT\ln\varphi_{k\left(aq\right)}=RT\ln x_{k\left(aq\right)}-RT\ln{\overset{˘}{x}}_{k\left(aq\right)}$$

(A9) $$\ln\varphi_{k\left(aq\right)}=\ln\left(x_{k\left(aq\right)}/{\overset{˘}{x}}_{k\left(aq\right)}\right)$$

As a result of micellar solubilization is ${\overset{˘}{x}}_{k\left(aq\right)}>x_{k\left(aq\right)}$ follows $\ln\varphi_{k\left(aq\right)}<0$. With an increase in the total concentration of polymeric surfactant (above the critical micellar concentration), ${\overset{˘}{x}}_{k\left(aq\right)}$ increases ($x_{k\left(aq\right)}=const.$), while $\ln\varphi_{k\left(aq\right)}$ decreases, becoming increasingly negative.
